# Artisanal Gem Mining in Brazil: A Source of Genotoxicity and Exposure to Toxic Elements

**DOI:** 10.3390/ijerph20032510

**Published:** 2023-01-31

**Authors:** Ana Paula Rufino Santos, Lucas Zeferino Silva, Bruna Moreira Freire, Márcia Cristina da Silva Faria, Bruno Lemos Batista, Bruno Alves Rocha, Fernando Barbosa, Jairo Lisboa Rodrigues

**Affiliations:** 1 Instituto de Ciência, Engenharia e Tecnologia, Universidade Federal dos Vales do Jequitinhonha e Mucuri, Teófilo Otoni 39803-371, MG, Brazil; 2 Centro de Ciências Naturais e Humanas, Universidade Federal do ABC, Santo André 09210-580, SP, Brazil; 3 Analytical and System Toxicology Laboratory, Department of Clinical Analyses, Toxicology and Food Sciences, School of Pharmaceutical Sciences of Ribeirao Preto, University of Sao Paulo, Avenida do Cafe s/no, Ribeirao Preto 14040-903, SP, Brazil

**Keywords:** miners, environmental contamination, occupational exposure, genotoxicity, biomonitoring

## Abstract

Environmental and occupational exposure to toxic metals has led many people around the world to have serious health problems. Mining activities contribute to an increased risk of exposure to these elements. In this work, a study of environmental biomonitoring and routes of exposure to toxic metals in a region of artisanal mining was performed. This study was carried out in the district of Taquaral de Minas, located in the Jequitinhonha Valley in the state of Minas Gerais. The valley is one of the wealthiest and highest gem-producing areas in Brazil. Five artisanal mines were sampled (Bode, Pirineu, Pinheira, Lajedo, and Marmita). Several potentially toxic metals (Be, Zn, Mn, Ba Cd, Hg, and U) were investigated in the soils and dust over the rocks and the soils. Samples from 22 individuals occupationally exposed and 17 unexposed persons, who formed the reference group, were analyzed for trace elements by an inductively coupled plasma mass spectrometer. The genotoxicity was evaluated by the micronucleus test in buccal mucosa epithelial cells, where the following changes were scored: micronuclei (MN) binucleate (BN) cells and kariolytic (KL) cells. The MN test showed significantly increased frequencies in all alterations of exposed individuals compared to the controls (*p* < 0.05, Student’s *t*-test). The urine analysis showed levels of Cr, Ni Ba, Pb, and As in the blood, which were higher than the ATSDR recommended levels. The association between the MN test and the trace element concentrations found in the blood and urine was significant (*p* < 0.05). The higher the number of years of working, the higher the concentrations in the blood were, due to chronic exposure. The results of the present study indicate environmental contamination and a potential risk to the health of miners, suggesting an intervention.

## 1. Introduction

In many regions of Brazil [1] and the world [2,3], the search for gems and other mineral rarities is the only option for survival for millions of manual workers; however, it is a degrading activity for human health and the environment [1]. Jequitinhonha Valley is among the different regions in Brazil that exhibit this extensive occupational exposure. This Valley is classified by the Mineral Resources Research Company [4] as one of the most important global gem-producing areas, which includes the cities of Virgem da Lapa, Rubelita, Coronel Murta, Itinga Medina, and Pedra Azul (State of Minas Gerais, Brazil). The region is known worldwide for producing several gemstones of important occurrences, such as tourmaline, beryl, tin (cassiterite), feldspar, lithium (amblygonite, spodumene, and petalite), mica, niobium-tantalum, and quartz [4].

Mining processes generally release metals and non-metals immobilized in rocks, sediments, and soil into the environment [5]. The higher the availability, the higher the toxicity and the higher the chance of bioaccumulation in living organisms [6]. In this context, exposure to chemicals may cause toxic effects and changes in the health status of individuals living in or in contact with such environments [7].

Several studies reported that, in the world, workers performing an activity might be exposed to an aggressive agent, with immediate effects such as accidents or occupational chronic diseases [8,9,10]. The toxic effects of exposure of workers to metals from artisanal mines can be classified as acute and chronic intoxication [11].

The objective of occupational toxicology is to manage and understand the various chemical risks present in workplaces [12]. Ferreira and Wermelinger (2013) [7] reported that human beings are exposed to different chemical, physical, and biological agents, whose exposures arise from contact through the respiratory, dermal, or ingestion routes. All metals and their compounds present toxicity depending on the degree, amount, and time of exposure [5]. Therefore, human exposure to these diversified chemical substances leads to the development of deleterious effects, including damage to the ecosystem, such as neurotoxicity, carcinogenic, mutagenic effects, and others [13]. For example, mutagenic effects occur when an agent can cause sudden changes to genetic material, and this effect is persistent and permanent in its structure or content [14], in addition to several other problems for the worker’s life. In contrast, a compound that causes metabolic alteration in cells, culminating in death or otherwise, presents cytotoxicity [15]. In this sense, the genotoxic agents can damage a DNA molecule, which is usually associated with situations of environmental pollution, such as gem mining [16]. Thus, it is extremely important to have studies that can assess the effects of artisanal mining on populations around the world.

Therefore, this research aimed to evaluate the occupational miners exposed in artisanal mines to environmental components (soil, soil dust, and stone dust). Moreover, toxicological parameters (blood and urine analysis, and genotoxicity by the micronucleus) were investigated.

## 2. Materials and Methods

### 2.1. Studied Area

**Figure 1:**
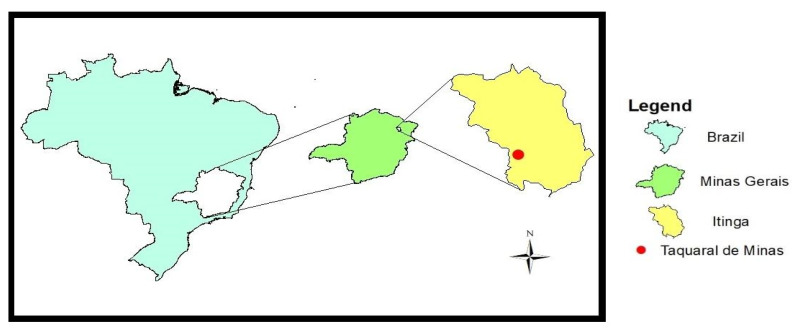
Location of biological and environmental samples (district of Taquaral de Minas, municipality of Itinga, state of Minas Gerais, Brazil). Geographic coordinates 41°51′45″ W. 16°42′ 45″ S.

The district of Taquaral de Minas is located in the municipality of Itinga, southwest of the district, in the Jequitinhonha mesoregion, northeast of the state of Minas Gerais (Brazil). In geological terms, Taquaral de Minas is a district with an abundance and variety of gems in the Eastern Pegmatitic Province of Brazil (Figure 1). This area has an estimated total population of 2364 inhabitants, as shown in the Epidemiological Map (created in 2014 at the Family Health Station in Taquaral de Minas, Minas Gerais, Brazil).

The Eastern Pegmatitic Province of Brazil extends from the northeast to east of Minas Gerais, along the valleys of the Doce and Jequitinhonha Rivers, where their main rocky bodies appear. This district has an abundance of rare chemical elements, such as lithium and boron in pegmatites, which, through the changes generated by the geological processes, have become deposits with a large number of valuable minerals [17].

### 2.2. Studied Population

The studied population consisted of 22 workers from areas of artisanal gem mining in the district of Taquaral de Minas, in the municipality of Itinga in the state of Minas Gerais (Brazil). The control group was composed of 17 individuals with no involvement in any occupational (mining) activity, including students, administrative staff, and docents of the Institute of Science, Engineering, and Technology (ICET) of the Federal University of the Valleys of Jequitinhonha and Mucuri (UFVJM). The general characteristics of the populations studied are summarized in Table 1.

Recruitment of exposed workers was carried out by contacting a public establishment in the city, where community health agents provided information and invited workers to participate in the study. This study was approved by the Research Ethics Committee of the Federal University of Jequitinhonha and Mucuri valleys (UFVJM), in accordance with opinion 3,692,758 and CAAE: 22164919.0.0000.5108. All participants were informed about the study and signed an informed consent form.

The existence of the mines in the Jequitinhonha Valley has historical significance, through the creation and settlement of several cities. This is what happened in Taquaral de Minas, a settlement that emerged on the banks of the Jequitinhonha River due to the discovery and extraction of gems [18]. Among the mineral resources extracted from the 30 mines around Taquaral de Minas, tourmaline gemstones are highlighted [1].

### 2.3. Analytical Instrumentation

All the trace elements were determined by an inductively coupled plasma Mass spectrometer (ICP-MS). The ICP-MS is a relatively new and effective technique for multi-element determinations, with very low interference and excellent limits of detection, and is ideal for trace analysis in water, biological samples (blood, urine, hair, and fingernails), and soils/sediments. A Perkin Elmer Nexion 300D (New York, NY, USA) instrument was used with a Meinhard nebulizer and cyclonic spray chamber and continuous nebulization. The operating conditions were: (i) nebulizer gas flow rates of 0.95 L min^−1^; auxiliary gas flow of 1.2 L min^−1^; (ii) plasma gas flow of 15 L min^−1^; (iii) lens voltage of 7.25 V; (iv) ICP RF Power of 1200 W; and (v) CeO/Ce ≤ 0.04%.

### 2.4. Reagents

Nitric acid was previously purified by sub-boiling distillation using a Savillex DST 1000 (Shelton, GA, USA). The Type I water (resistivity 18.2 MΩ cm^−1^) was obtained by a Thermo Scientific Barnstead Nanopure purification system. The solutions were prepared daily, and all analyses were performed in the Laboratory of Analytical Instrumentation at the Federal University of Jequitinhonha and Mucuri Valley (UFVJM, Mucuri Campus), a Class 1000 clean room. Quality control for the determination of metals was carried out by the analysis of standard reference materials. There were no statistical differences between the concentration values obtained for the reference materials, and the “target values” at 95% confidence intervals using the *t*-test.

### 2.5. Sampling of Soil, Soil Dust, and Stone Dust

The soil, soil dust, and stone dust samples of the rocks inside the underground mines were collected in October 2016 at five specific mining sites—namely, Bode, Pirineu, Pinheira, Lajedo, and Marmita. The determination of the metals in the dust samples superimposed on the underground extraction environment followed the guidelines proposed by Ono et al. (2011) [19]. First, a brush with bristles of nylon (18 cm long) was used (depths of 0–10 cm) to remove the soil dust and stone dust. Soil sampling was performed using a manual excavation tool (depth of 0 to 20 cm). The samples were homogenized and stored in a soil-collecting bag (NASCO brand, Brazil). Three samples of each artisanal mine were collected at specific points: two of the dust samples were collected from the mines’ soils and one from rock walls. The plastic bags were labeled and sealed at the end of the sampling.

### 2.6. Preparation and Analysis of Environmental Samples

The samples were air-dried and macerated (grade and pistil). A 150 μm nylon sieve was used for sample sieving. Acid extraction was carried out using the US EPA 3051. A method of the US Environmental Protection Agency (USEPA) in a microwave-assisted sample digestion system MARS-6^®^ (910900, CEM, Matthews, NC, USA). For this procedure, 0.5 g of solid material was added to a Teflon flask with 10 mL of previously purified (sub-boiled) HNO_3_. After digestion, the samples were transferred to a Falcon tube, reaching its maximum capacity, 15 mL, with Type I water. For analysis, 0.1 mL of the supernatant was diluted 1000-fold with 2% (*v*/*v*) HNO_3_ for multielement determination by ICP-MS.

### 2.7. Sampling, Preparation, and Analysis of Buccal Exfoliative Cells

The individual samples were collected for evaluation of human exposure after approval from the Research Ethics Committee of UFVJM (protocol number 1,691,988). Research volunteers collected signatures from the participants for their free participation and informed consent. Some details of the study are also present in the UFVJM master’s dissertation collection [20].

The collection of buccal cells followed the protocol proposed by Thomas et al. (2009), [21]. The method consists of scraping the buccal mucosa using a wooden tongue depressor. The material was transferred into falcon tubes containing 10 mL of buccal buffer (Tris—Sigma Aldrich, St. Louis, MO, USA; EDTA—Sigma Aldrich, USA; sodium chloride—Sigma Aldrich, USA) and stirred (vortex Thermo Scientific, M37615, Waltham, MA, USA).

The cells were placed in a centrifuge (Cetec 6000R, Ribeirão Preto, SP, Brazil) and centrifuged for 10 min at 581× *g* at 25 °C. This procedure was repeated three times. About 120–150 µL of the cell suspension was transferred to the air-dried slides and fixed in Carnoy solution containing ethanol (Neon, Suzano, Brazil) and glacial acetic acid (Isofar, Duque de Caxias, Brazil) (3:1). For staining, the slides were dipped for 1 min each into flasks containing 50% (*v*/*v*) and 20% (*v*/*v*) ethanol and washed for 2 min in a vessel containing Type I water. After that, they were placed in a vial of 5 M hydrochloric acid (Isofar, Brazil) for 30 min and then rinsed in tap water for 3 min. Schiff’s reagent (Sigma Alcrich, St. Louis, MO, USA) was added for core staining (approximately 1 h, in an environment without light.). For the counterstaining of the cytoplasm, Fast-Green (Sigma Aldrich dye, St. Louis, MO, USA) was used for 20–30 s. The analysis occurred at a magnification of 400 and 1000 times under an optical microscope. The scoring criteria followed those described by Thomas et al. (2009) [21] in a minimum of 2000 differentiated to the micronucleus and 1000 other cell modifications (shoots, binucleate, karyiorrex, cariolytic, picnotic).

### 2.8. Sampling, Preparation, and Analysis of Biological Specimens (Blood and Urine)

A nurse collected the venous blood samples (4 mL) of the volunteers in a private room of a clinical laboratory. Before the venous puncture, the skin was cleaned with 70% alcohol. The blood was collected in vacuum tubes for trace elements (BD, Vacutainer^®^, Franklin Lakes, NJ, USA) containing anticoagulant (EDTA). They were transported at −20 °C and stored at −80 °C until analysis. The analyses of the urine and blood were carried out as described elsewhere [20]. All samples were prepared using a diluent containing distilled HNO_3_ 0.3% *v*/*v* and Triton X-100 0.002% *m*/*v*. For analysis, the samples of blood and urine were diluted by 1 + 49 and 1 + 19, respectively. All analytical calibrations were from 1 to 20 µg/L, except for Hg, for which the calibration ranged from 100 to 1000 ng/L. The reference materials analyzed for quality assurance in the trace element determinations were the whole blood Seronorm L-2 (Sero, Billingstad, Norway) and the freeze-dried urine NIST 2670a (National Institute of Standard and Technology, Gaithersburg, MD, USA).

### 2.9. Statistical Analysis

The concentrations of the chemical elements in the environmental samples were compared with the Guiding Values for Soil and Groundwater (Quality Reference Value—QRV and Prevention Value—PV) presented in the most recent publication of the Official Gazette of the State of São Paulo, the Decision of the Board of Directors 045/2014 of CETESB, the National Environmental Council (CONAMA), and the Normative Deliberation of COPAM (Specific Council of Environmental Policy) 166/2011, specific to the state of Minas Gerais, in addition to the ATSDR International Resolution (Agency for Toxic Substances and Disease Registry) for the year 2002.

The descriptive statistics (mean and standard deviation) were used for the soil and dust/soil and rock samples. Microsoft Excel^®^ (Microsoft Office 365, Washington, DC, USA), was used for statistical analyses. For the components obtained by the micronucleus assay, descriptive data regarding age and habits (such as tobacco use and alcohol consumption) were presented in percentages.

A paired *t*-test (significance at *p* < 0.05) was used for the comparison of commonalities associated with changes in the exposed individuals and controls and for the concentrations of chemical elements. The blood, urine, and creatinine results were expressed as the minimum and maximum intervals, mean, median, and the 10th, 25th, 75th, and 90th percentiles. All statistical analyses were enhanced using GraphPad Prism Software (GraphPad, Version 8.0, La Jolla, CA, USA).

## 3. Results and Discussion

### 3.1. Concentration of Chemical Elements in Environmental Samples

Chemical elements are widespread, and some are considered essential micronutrients for plants. Other elements occur at low values in soils, depending on the composition of the source material, soil development and formation processes, and the environmental characteristics of the area [22].

Although they are associated with toxic capacity, a number of metals are in the constitution of rocks and plants and have a natural and beneficial occurrence in the environment, including Fe, Mn, Ni, Cu, Zn, Mo, and Co. On the other hand, Pb, Cd, and Hg are potentially toxic without any essentiality to soils or living organisms [23]. Such elements, at high concentrations, can cause miscarriages, neurological malformation, and cancers (skin, pancreas, and lung) [24].

In this study, analyses of the elements Li, Be, V, Cr, Mn, Ni, Cu, Zn As, Sr, Cd, Ba, Pb, Bi, U, and Hg were carried out in 15 samples of soil and dust in the artisanal mines visited (Bode, Pirineu, Pinheira, Lajedo, and Marmita). The results are the means of triplicate readings of each sample. The Supplementary Materials (Tables S1 and S2) present the concentrations of the metals in the samples of soil, soil/rock dust, and dust. In addition, there is a comparison with the values established by CETESB (2014) [25], COPAM (2011) [26] and ATSDR (2002) [27], respectively. Only the elements that obtained significant changes (Ba, Be Cd, Hg, Zn, U, and Mn) are discussed below.

According to the Resolution of COPAM 166/2011 [26] and CETESB (2014) [25] the elements Ba, Cd, Hg, and Zn presented values above the recommended limits. According to the Agency for Toxic Substances and Disease Registry (ATSDR, 2002) [27] Be, Mn, Zn, and U exceeded the established limit. The other elements were within the regulated limits.

The soil collected in the Marmita mine (Table S1) presented Ba levels (104 mg/kg) higher than the recommended limits from CETESB and COPAM (75 and 93 mg/kg, respectively). The elements Cd, As, and Hg also exceed the recommended limits. The higher Hg concentrations were from past gold mining and incorrect waste management. Mercury was in the environment in different components (soil, soil dust, and stone dust; Table S1) [28].

The presence of Cd occurs due to several factors, such as weathering, soil erosion, landfill leaks, and mining residues. Cadmium is chemically similar to Zn, and both are usually found together in geochemical processes [29]. This fact may explain the higher concentrations of these elements in the prospected mines. In relation to Zn, the soils sampled showed high concentrations in the mines of Bode, Pirineu, and Marmita. Zinc also showed high concentrations in the dust/soil dust from the Bode mine. Amorim (2012) [30] reported that, although Zn is an abundant and non-toxic element, anthropogenic activities, such as mining, bring environmental concerns and legal responsibility.

Berylium, Mn, and U are not regulated by COPAM or CETESB. Regarding Be, the concentrations in the soil from Pirineu, in the soil dust from Pirineu, Pinheira, Lajedo, and Marmita, and in the stone dust from Bode, Pirineu, Lajedo, and Marmita presented concentrations higher than 15 mg/kg, as established by ATSDR (2002) [27].

Biondi (2010) [23] described the relevance of the determination of natural Mn concentrations in soils worldwide, since it is the main constituent of rocks and plant micronutrients [22,31]. The values of the element Mn were higher than that recommended (330 mg/kg) by ATSDR (2002) [27] at the mines of Pirineu, Pinheira, Lajedo, and Marmita (soil); Pirineu, Pinheira, and Marmita (soil dust); and Bode, Pirineu, Pinheira, and Marmita (stone dust).

Uranium is a natural component of soil and rocks that can be transferred to the air by mining processes. The main route of exposure to the element is oral, followed by inhalation [27]). In all samples, U presented concentrations higher than 3 mg/kg, according to ATSDR (2002) [27] (Table S2).

The concentrations of Al ranged from 4336 mg/kg to 13,660 mg/kg, with an average value of 8127 mg/kg. In Brazil, there are still no reference values for Al in soils. By comparison, the presented concentrations were lower than the minimum concentration found (17,770 mg/kg) by Silva (2011) [32] in the eight basins of the rivers Velhas, Paracatu, Abaeté, Urucuia, Carinhanha, Jequitaí, Verde Grande, and São Francisco in the state of Minas Gerais (Brazil). They reported that Al is present in a series of minerals, such as alunite, andalusite, beryl, biotite, kyanite, cordierite, spodumene, staurolite, muscovite, feldspar, and sillimanite. In this way, synergism can happen between its natural occurrence and occupational exposure. Therefore, due to all these concerns, other variables should be considered, such as age, gender, diet, family characteristics, lifestyle, health status, and the dose, time, and method of exposure.

### 3.2. Determination oMf Genotoxic Changes in Exposed Individuals and Controls

**Figure 2:**
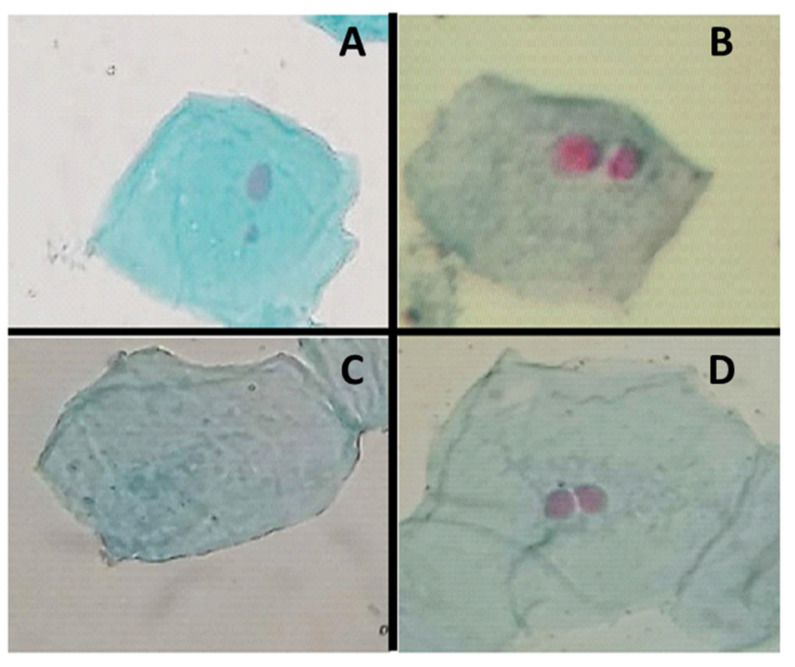
Alterations observed in exposed individuals and controls. (**A**): Micronucleus cell (MN); (**B**): Binucleated cell (BN); (**C**): Karyolitic cell (KL); (**D**): Binucleated cell (BN).

In humans, the micronucleus test (MN) can be easily assessed in exfoliated epithelial cells of either the buccal or nasal epithelium. This test is conducted to measure genetic/genomic damage in vivo in occupational/accidental exposure or for the assessment of lifestyles, cancer detection, or neurodegenerative diseases [33,34]. The efficacy of the MN test on buccal epithelial cells has been recognized by other studies [35] for the detection of genotoxic effects or exposure to mutagens. Nuclear abnormalities or additional biomarkers, such as binucleated (BN), karyorrhexis (KR), pyknotic nuclei (PN), karyolitic cell (KL), condensed chromatin (CC), and the micronucleus, can be identified by the MN test during cell differentiation, which indicates damage to DNA, cytotoxicity, or cell death when observed at high levels [36,37]. Figure 2 shows the cellular alterations found in the studied groups. Figure 2 shows the distribution of changes in the controls and the exposed participants.

**Figure 3:**
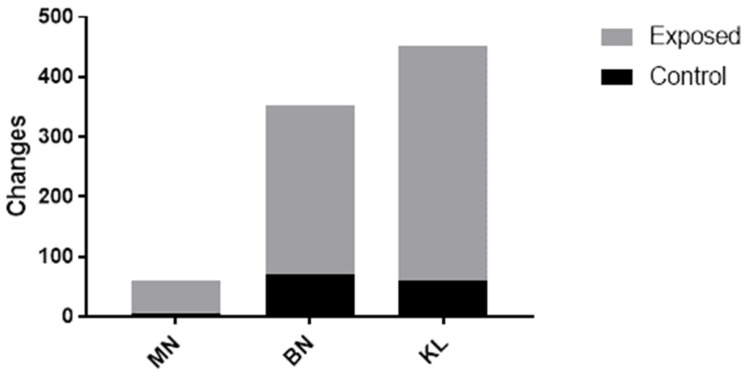
Distribution of nuclear abnormalities of the control (black) and exposed (gray) groups. *p* < 0.05 according to the sample paired *t*-test.

The analysis of the genotoxicity biomarkers showed significant differences (*p* < 0.05) between the populations in the frequency of MN, BN, and KL. The higher values were found for the exposed group. The results in Figure 3 (and Supplementary Materials Table S3) are similar to those found by Arul et al. (2017) [38] and Coelho et al. (2011) [39], who aimed to determine the adverse health effects on populations living in the vicinity of mining regions (As and Cd exposure). Table 2 shows the influences between the habits of the studied groups and the frequency of chromosomal abnormalities. Castañeda-Yslas et al. (2016) [40], intending to evaluate the genotoxic effect of the use of pesticides on the buccal mucosa in farmers and their children, concluded that a high number of MN and nuclear abnormalities (NA) might indicate future health damage. Table 2 shows the influences of the habits of the studied groups on the frequency of NA.

The significant number of changes found in occupationally exposed individuals may indicate genotoxic damage induced by chronic exposure and possible metal contamination. Baimain et al. (2003) [41] reported that genetic alterations are significant in the development of cancer, since most cancer cells present genomic instability. Inherited mutations may induce such instability in genes or those acquired in somatic cells in the development of a tumor.

Bolognesi et al. (2013) [42] indicated that the presence of MN and BN in occupationally exposed groups reflected genotoxic damage and cell death. On the other hand, PN, CC, KR, and KL occurred after cytotoxic damages. The formation of DNA damage by exposure to chemicals has been associated with the occurrence of chronic and degenerative diseases. BN cells, for example, may indicate a failure in the cytokinesis process, MN chromosomal instability, or DNA damage, while other changes, including CC, KR, PN, and KL, indicate cell death.

**Figure 4:**
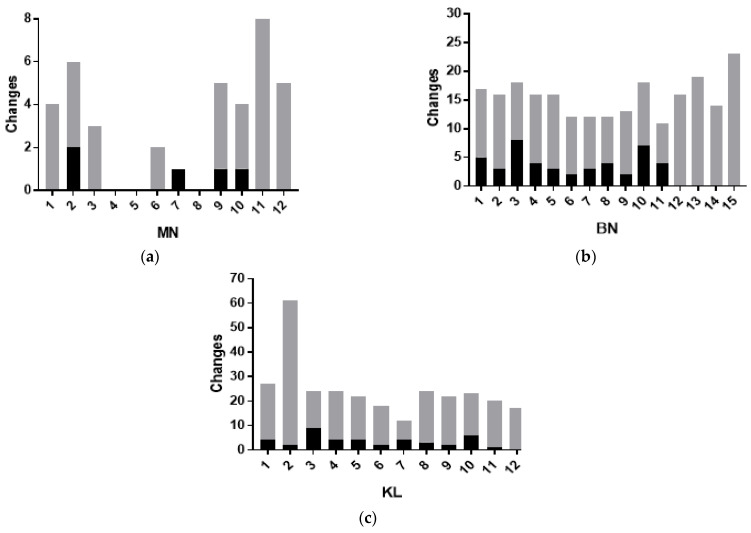
Influence of alcohol consumption on the distribution in the control (black) and exposed groups (gray). *p* < 0.05 according to the sample paired *t*-test. (**a**) Micronucleus (MN), (**b**) binucleated (BN), and (**c**) karyolitic (KL).

The results of this study show significant differences (*p* < 0.05) related to the consumption of alcoholic beverages between the controls and those exposed to MN, BN, and KL cells (Figure 4). However, the absence of ethanol consumption did not influence or interfere with the frequency of changes. Based on the analysis of Figure 4, differences between the frequency of changes in the studied groups were observed. The KL cells of exposed individuals presented increased frequency compared to BN and MN cells (Figure 2). In the controls, the increase was in BN, followed by KL and MN, respectively.

Considering the group with reported alcoholic beverage consumption, approximately 67% (8 exposed individuals) presented MN; for the control group, approximately 33% (4 subjects) presented MN. Approximately 73% (11 individuals) of the control group presented BN, but the frequency in the exposed group increased, occurring in 100% of the cases, a fact that was also observed in KL, whose values were similar.

Ethanol is considered an important chemical agent in the development of genotoxic damage to the oral mucosa. Even considering the high rate of cell renewal, changes can be observed in buccal exfoliative cells from subjects who reported the consumption of alcoholic beverages [43]. Stich (1988) [44] and Ghose and Parida (1995) [45] positively associated the increased frequency of MNs with individuals who consumed both alcoholic beverages and tobacco.

As observed, the results found for the controls and exposed individuals who smoked were not significant, since only one individual in the control group was a smoker and there were three in the exposed group (Table 2). Bonassi et al. (2003) [46] found that the frequency of MN in smokers was not statistically different from non-smokers who presented a high frequency of MN. In a comparison between studies (MEDLINE database) conducted on individuals exposed to genotoxins and their controls, the same author mentioned that smoking (33 publications; 89.2%) was not associated with MN [47]. On the other hand, Motgi (2014) [48] found differences between individuals with smoking habits compared to controls, which can trigger cytotoxic and genotoxic damages.

Other investigations in the literature support the importance of our findings, mainly when genotoxic tests are associated with biomonitoring studies. For instance, Wegner et al. (2000) [49] aimed to ascertain the degree of occupational exposure to Be in miners of marine water and emerald extraction areas in Germany. Cheyns et al. (2014) [9] in turn, investigated the level of human and environmental exposure to Co in Africa by analyzing dust, contaminated soils, and urine. Coelho (20112) [10] evaluated the adverse health effects of metal contamination by biomonitoring biological specimens and the MN in Portugal. Joca (2009) [50] evaluated the genotoxicity in workers exposed to silica in Brazil. Other studies, such as Paruchuri et al. (2010) [51], Steckling et al. (2011) [52] and Molina-Villalba et al. (2015) [53] presented results that were related to occupational exposure in mining areas.

### 3.3. Determination of Trace Elements in Blood and Urine

Firstly, creatinine adjustment is routinely used to reduce factors that are not directly related to metal exposure, such as the concentration and volume of urine [54]. The urine results were adjusted and plotted as micrograms of metal per gram of creatinine.

As far as we know, this study is the first to analyze the possible occupational exposition by bioindicators (urine and blood) existing in the individuals who work in the Jequitinhonha Valley gemstone exploration in Minas Gerais (MG). Twenty metals (Li, Be, Al, Ca, Cr, Mn, Fe, Co, Ni, Cu, Zn, As, Se, Sr, Cd, Cs, Ba, Hg, Pb, and U) were quantified in the blood and urine of the exposed population (22) and control individuals (17). The Supplementary Materials (Tables S4–S8) show the values of the concentrations found, which are described as the minimum and maximum, mean, median, and the 10th, 25th, 75th, and 90th percentiles for the blood and urine of the exposed group and control group.

The elimination of trace metals occurs through the kidneys and the gastrointestinal tract, which takes time, depending on the element. The period for which half of the initial bioaccumulated amount is excreted varies widely. Lead and Cd take from 10 to 12 years; As takes about 4 days; and Hg takes 60 days. Therefore, blood and urine generally reflect recent exposure, including days and weeks, and other matrices, such as nails, may reflect exposure for months [55]. Hall and Guyton (2011) [56] showed that urine functions as a mechanism in the human body to excrete water-soluble contaminants, excess water, and a variety of toxic substances and metals. Although occupational exposure is a chronic and recurrent condition due to daily activity, the analyses of blood and urine samples are matched to suit the situation.

Comparing the urine results from the exposed group and the control, almost all the analyzed elements had higher mean values, except Se and Ce, whose means in the controls (31.31 and 22.73 μg L^−1^, respectively) were above those in the exposed group (19.72 and 22.27 μg L^−1^, respectively). For U, the values were below the detection limits.

The average results of the workers’ urine found for the other elements were: Li—30.42 µg/L; Be—0.25 µg/L; Al—33. 44 µg/L; Cr—0.38 µg/L; Mn—0.72 µg/L; Fe—19.65 µg/L; Co—0.64 µg/L; Ni—3.28 µg/L; Cu—59.80 µg/L; Zn—523.60 µg/L; As—20.44 µg/L; Sr—200.04 µg/L; Cd—0.18 µg/L; Ba—6.49 µg/L; Hg—3.74 µg/L; and Pb—2.79 µg/L.

The ATSDR [57,58,59] established reference values for some metals in urine and blood. Concerning urine, Be, Mn, Cd, and As were within the reference levels: 0.28 μg L^−1^, 1.19 μg L^−1^, 0.185 μg L^−1^, and less than 100 μg L^−1^, respectively. The ATSDR [60,61,62] averages for these elements are Cr—0.22 μg L^−1^, Ni—1–3 μg L^−1^, Ba—1.5 μg L^−1^, and Pb—0.66 μg L^−1^. Similarly, Al presented high mean levels (33.44 μg L^−1^).

The reference values for several elements in the urine were established by Batista et al. (2009) [63] in Brazil. For Al, the mean value was 3.4 μg L^−1^, with a minimum and maximum range of 0.22–17.5 μg L^−1^, for Ni; 1.3 μg/L was the mean value, with intervals of 0.1–4.2 μg/L; Ba had intervals of 0.2–5.3 μg/L and a mean of 1.5 μg/L; and the mean of Pb was 0.8, with intervals lower than 0, 03–2.96 μg/L. Compared to the results of the present study, all the mentioned elements exceeded the maximum values found: 115.77 μg/L; 8.55 μg/L, 30.37 μg/L; and 13.88 μg/L, for Al, Ni, Ba, and Pb, respectively.

An investigation carried out in the Araçuaí region (Minas Gerais, Brazil) found Al concentrations in plasma that indicated contamination and exposure of the population in some communities [32]. This element is of natural origin and its route of exposure is “rock–soil–water–food”.

Coelho et al. (2011) [39] investigated a deactivated mine in Panasqueira (Portugal). They identified the contamination of volunteers. The means found for As, Cd, Cr, Mn, Ni, Pb, and Se in the urine were 43.01, 0.83, 1.15, 1.36, 8.16, 4.54, and 31.15 μg/g. The results of the exposed individuals of the present study are below their concentrations, i.e. As—3.43 μg/g; Cd—0.03 μg/g; Cr—0.07 μg/g; Mn—0.23 μg/g; Ni—0.56 μg/g; Pb—0.48 μg/g; and Se—3.05 μg/g (Table S8).

Regarding blood, the highest values of the exposed group were found for Li, Ca, Mn, Fe, Co Ni, Sr, Cd, Ba, and Pb. On the other hand, for the controls, Ba, Al, Cr, Cu, Zn, As, Si, Cs, and Hg presented mean values higher than the values of the mine workers.

For Al, this situation can be explained by taking into account the irregular distribution of concentrations in the control group employing the percentile analyses. When verifying the mean and maximum values obtained for the element in the exposed group (3.66 μg/L and 23.34 μg/L), the values are significantly lower than those obtained for the controls (7.42 μg/L and 123.43 μg/L). However, in 90% of cases, the value found was 1.07 μg/L, which was relatively discrepant for both the mean and maximum values presented. This fact explains that one person was responsible for raising the element’s mean. For Cr, the average value was 0.77 μg/L and the maximum was 10.73 μg/L, while, in the 90th percentile, the value was 0.90 μg/L. Mercury in the control group presented a mean of 0.96 μg/L, a maximum of 3.32 μg/L, and the 90th percentile was 1.73 μg/L.

Other elements of the samples from the exposed group for As, Cd, Cu, Mn, Ni, Pb, and Se were 1.45 μg/L, 0.14 μg/L, 1062.64 μg/L, 12.85 μg/L, 15.27 μg/L, 74.26 μg/L, and 53.79 μg/L. When compared to the results obtained by Nunes (2010) [64] (1.1 μg/L, 0.4 μg/L, 890 μg/L, 9.6 μg/L, 2.1 μg/L, 65, 4 μg/L, and 89.3 μg/L for As, Cd, Cu, Mn, Ni, Pb, and Se, respectively), the elements Cd and Se are within what is proposed by the author, while As, Cu, Mn, Ni, and Pb presented higher values.

Among the reference limits reported by the ATSDR [57,58,59], the values of blood Cd (0.315 μg/L) were above the maximum values, and blood As (<1 μg/L) were below the limits.

Rodrigues et al. (2009) [65] carried out a study in the Amazonian riverside population (a region near a mining area), in which the values found for Cu 920 μg/L and Co 0.40 μg/L were below those identified in Taquaral de Minas (means of 1062.63 μg/L and 0.44 μg/L, respectively). The concentrations of the present study were higher than the maximum and minimum intervals of the elements Mn (5.1–14.7 µg/L), Cu (494.9–2383.8 µg/L), and Pb (5.9–330.1 µg/L) reported by Rodrigues et al. (2008) [66]. On the other hand, for Mn, Freire et al. (2015) [67] reported 12.85 μg/L for metal workers.

Observing the results from Coelho et al. (2011) [39], the means of the elements Pb, Mn, Se, and Ni were 4.54; 1.36; 31.15, and 8.16 μg/L, respectively. For Hg, the mean of the population in the metropolitan area of São Paulo was 66.00 μg/L [68]. The ATSDR [61] recommends a mean Pb value of 1.5 μg dL^−1^, which is lower than that of 7.42 μg dL^−1^ found in this study [69].

The analysis of the metal quantification showed significant differences (*p* < 0.05) between the studied groups when compared to the control group for the two matrices studied.

The analyses of urine revealed statistical differences for the following elements: Li, Be, Al, Mn, Cu, Cd, Hg, and Pb. In blood, therefore, seven elements (Li, Mn, As, Se, Sr, Cs, and Pb) had significantly increased concentrations (Table 3).

The results of the age groups and working times related to the concentration of the elements are present in Tables S9–S12. The distribution of urine and blood in the age intervals occurred regularly. The concentrations of elements with high values in the urine occurred between 60–70 years, for Li, Fe, Co, Ni, Cd, and Pb. In blood, these values were observed in the same age group for Li, Al, Co, Cu, Sr, Cd, and Ba.

By establishing an association between the working time in the mine and high concentrations in the urine, higher frequencies of Li, Be, Fe, Ba, and Pb were observed with 20–30 years of work. In the blood, these values were concentrated in the time range between 30 and 40 years for Al, Cr, Ni, Co, Mn, Sr, Cd, and Ba. This result indicates that miners with longer working hours had higher blood concentrations, due to possible chronic exposure. The three miners within the age range of 60–70 years had been performing mining activity for more than 30 years, confirming that prolonged exposure and the age factor led to an increase in the metal concentration in these bioindicators.

## 4. Conclusions

The present study indicates that miners are exposed both environmentally and occupationally. The high concentrations of the metals, especially the toxins found in the environmental samples (Ba, Cd, Hg, U), are capable of causing effects on human health. The dust found in the underground mines and superimposed on soil or rocks is in direct contact with the dermal and ingestion pathways of workers. This situation tends to promote genotoxic effects, such as those observed through the significant increase in the number of alterations (MN, BN, and KL) found when compared to the control individuals. Biomonitoring analyses showed levels of Cr, Ni Ba, and Pb in the urine and As and Pb in the blood that were higher than the ATSDR reference values. The association between age groups and working time in gem extraction activities showed that miners with longer working hours had higher concentrations of potentially toxic chemical elements in the blood, likely due to chronic exposure.

This study is fundamental for the biomonitoring of an exposed population, which is generally ignored by authorities, and the stimulation of similar investigations in other areas of artisanal mining. The development of preventive or remediation measures, through the evaluation and understanding of the direct relation obtained by exposure and the subsequent biological effect, may lead to a reduction in health damage or cancer risk in humans. Therefore, the current results are essential for conducting new environmental and biological monitoring activities in mineral exploration areas.

## Figures and Tables

**Table 1:** Characteristics of the studied populations.

	Control Group	Occupationally Exposed Group
Number of subjects	17	22
Age (years, mean ± SD)	33.23 ± 7.54	53.68 ± 12.28
(Range)	(23–48)	(26–77)
Smoking habits		
Nonsmokers, N, (%)	16 (94.12)	19 (86.36)
Smokers, N (%)	1 (5.88)	3 (13.64)
Alcohol consumption		
Positive, N (%)	11 (64.70)	12 (54.55)
Negative, N (%)	6 (35.30)	10 (45.45)

**Table 2:** Influence of age, alcohol consumption, and smoking on the genotoxicity biomarkers studied.

Alteration Frequencies of Groups	*p* Value (*t*-Test)
Age Control Group × Age Exposed	0.1072
MN Control Group × MN Exposed	0.0071 *
BN Control Group × BN Exposed	0.0004 *
KL Control Group × KL Exposed	0.0003 *
Alcoholic Consumption:	
MN Control Group × MN Exposed	0.0040 *
BN Control Group × BN Exposed	0.0001 *
KL Control Group × KL Exposed	0.0092 *
Non-alcoholic Consumption:	
MN Control Group × MN Exposed	0.1184
BN Control Group × BN Exposed	0.0524
KL Control Group × KL Exposed	0.0559
Smokers	
MN Control Group × MN Exposed	0.3019
BN Control Group × BN Exposed	0.3063
KL Control Group × KL Exposed	0.2438
Nonsmokers	
MN Control Group × MN Exposed	0.0010 *
BN Control Group × BN Exposed	0.0001 *
KL Control Group × KL Exposed	0.0001 *

* Indicates significant difference from control, *p* < 0.05.

**Table 3:** Comparison between urine and blood samples from the control and exposed groups (*p* values).

Element	*p* Value (*t*-Test)	
	Urine Samples	Blood Samples
Li	0.0146 *	0.0284 *
Be	0.0026 *	0.6997
Al	0.0126 *	0.5825
Ca	0.1918	0.3862
Cr	0.0001	0.3264
Mn	0.0040 *	0.0295 *
Fe	0.0703	0.9234
Co	0.1675	0.0499 *
Ni	0.0855	0.4206
Cu	<0.0001 *	0.7481
Zn	0.8304	0.8301
As	0.0802	0.0014 *
Se	0.0575	0.0002 *
Sr	0.7818	0.0005 *
Cd	0.0018 *	0.4237
Cs	0.9945	<0.0001 *
Ba	0.3654	0.1881
Hg	0.0043 *	0.9579
Pb	0.0063 *	<0.0001 *

* Indicates significant difference from control, *p* < 0.05.

## Data Availability

The datasets generated during the current study are available from authors upon reasonable request.

## Supplementary Materials

The following supporting information can be downloaded at: https://www.mdpi.com/article/10.3390/ijerph20032510/s1. Table S1. Concentrations of trace elements (mg kg^−1^) in the analyzed samples (soil, soil dust, and stonedust) and comparison with reference values (mean ± SD); Table S2. Concentration of trace elements (mg kg^−1^) in the analyzed soil samples and comparison with reference values (mean ± SD); Table S3. Frequency of MN, BN, and KL in control and occupationally exposed groups (Mean ± SD, Range); Table S4. Concentration (µg L^−1^) of chemical elements in urine of exposed group (minimum, maximum, mean, median and percentiles 10%, 25%, 75%, and 90%); Table S5. Concentration (µg L^−1^) of chemical elements in urine of control group (minimum, maximum, mean, median and percentiles 10%, 25%, 75%, and 90%); Table S6. Blood concentration (µg L^−1^) of chemical elements in exposed group (minimum, maximum, mean, median and percentiles 10%, 25%, 75%, and 90%); Table S7. Blood concentration (µg L^−1^) of chemical elements in control group (minimum, maximum, mean, median and percentiles 10%, 25%, 75%, and 90%); Table S8. Concentrations (μg g^−1^ creatinine) of chemical elements in urinary; Table S9. Relation (µg L^−1^) between elements in miners’ urine and age groups; Table S10. Relation (µg L^−1^) between elements in miners’ blood and age groups; Table S11. Relation (µg L^−1^) between elements in miners’ urine and working time; Table S12. Relation (µg L^−1^) between elements in miners’ blood and working time.
