# Supplementary material for: Artisanal Gem Mining in Brazil: A Source of Genotoxicity and Exposure to Toxic Elements
Source: Int J Environ Res Public Health. 2023 Jan 31;20(3):2510. doi: 10.3390/ijerph20032510 (PMC9916162; doi:10.3390/ijerph20032510)
Supplement: Supplementary file 1 [file ijerph-20-02510-s001.zip › ijerph-2179201-supplementary.pdf]

Table S1. Concentrations of trace elements (mg kg^{-1}) in the analyzed samples (soil, soil dust, and stonedust) and comparison with reference values (mean ± SD)

**Element**	**Mine**	**Soil**	**Soil dust**	**Stonedust**	**COPAM**		**CETESB**	
					**QRV**	**PV**	**QRV**	**PV**
Ba	Bode	32.22±0.38	1.00 ±0.10	0.16±0.13	93.00	150.00	75.00	120.00
	Pirineu	68.28±0.53	0.10±0.01	0.11±0.00				
	Pinheira	27.32±0.20	0.10±0.01	0.12±0.01				
	Lajedo	45.40±0.34	1.49±0.03	1.44±0.03				
	Marmita	104.18±0.88	5.30±0.04	11.70±0.03				
Cd	Bode	0.33±0.00	0.42±0.01	0.69±0.01	0.40	1.30	0.50	1.30
	Pirineu	0.53±0.01	0.85±0.03	0.72±0.01				
	Pinheira	0.33±0.00	0.81±0.02	0.50±0.01				
	Lajedo	0.45±0.01	0.73±0.01	0.52±0.01				
	Marmita	0.46±0.01	0.67±0.02	0.97±0.02				
Hg	Bode	133.81±18.77	1576.67±111.87	840.79±37.57	0.05	0.50	0.05	0.50
	Pirineu	180.15±40.67	339.04 ± 22.46	185.16 ± 26.24				
	Pinheira	92.32±28.94	916.18 ±64.66	401.63 ± 28.52				
	Lajedo	12.00±0.02	13.00±0.001	15.47 ± 0.01				
	Marmita	45.98±12.76	21.47 ± 0.02	8.21 ± 0.22				
Zn	Bode	61.50 ± 0.56	69.75 ± 0.92	4.79 ± 0.32	46.50	300.00	60.00	86.00
	Pirineu	78.25 ± 0.67	104.54 ± 1.01	94.16 ± 0.89				
	Pinheira	22.21 ± 0.29	82.36 ± 1.13	43.16 ± 0.59				
	Lajedo	23.24 ± 0.28	132.77 ± 1.28	33.98 ± 0.52				

Marmita	61.63 ± 0.83	36.50 ± 0.29	45.03 ± 0.38

*QRV/PV (Quality Reference Value/Prevention Value) COPAM and CETESB regulations.

Table S2. Concentration of trace elements (mg kg^{-1}) in the analyzed soil samples and comparison with reference values (mean ± SD)

Element	Mine	Soil	Soil dust	Stonedust	ATSDR<br>QRV
Be	Bode	2.74 ± 0.03	11.33 ± 0.47	20.7 ± 0.2	15.00
	Pirineu	41.3 ± 0.4	71.2 ± 1.1	28.6 ± 0.6	
	Pinheira	3.04 ± 0.05	26.94 ± 0.29	9.31 ± 0.11	
	Lajedo	5.33 ± 0.10	40.77 ± 0.92	38.37 ± 0.31	
	Marmita	4.76 ± 0.05	17.59 ± 0.52	34.62 ± 0.29	
Mn	Bode	161.00 ± 0.89	106.77 ± 1.29	504.95 ± 7.13	330
	Pirineu	958 ± 5.4	1257 ± 12.9	470 ± 4.7	
	Pinheira	539.31 ± 4.79	761.78 ± 11.53	395.56 ± 4.09	
	Lajedo	360.63 ± 2.25	185.09 ± 2.91	178.79 ± 2.54	
	Marmita	413.29 ± 3.12	707.50 ± 5.98	1600.49 ± 9.37	
U	Bode	19.33 ± 0.21	3.67 ± 0.01	4.90 ± 0.02	3
	Pirineu	58.09 ± 0.62	32.04 ± 0.34	4.61 ± 0.01	
	Pinheira	24.82 ± 0.31	12.08 ± 0.09	4.82 ± 0.01	
	Lajedo	19.88 ± 0.32	4.43 ± 0.02	4.31 ± 0.01	
	Marmita	102.00 ± 1.06	9.00 ± 0.03	13.56 ± 0.08	
Zn	Bode	61.50 ± 0.56	69.75 ± 0.92	4.79 ± 0.32	60
	Pirineu	78.25 ± 0.67	104.54 ± 1.01	94.16 ± 0.89	
	Pinheira	22.21 ± 0.29	82.36 ± 1.13	43.16 ± 0.59	
	Lajedo	23.24 ± 0.28	132.77 ± 1.28	33.98 ± 0.52	
	Marmita	61.63 ± 0.83	36.50 ± 0.29	45.03 ± 0.38	

*QRV (Quality Reference Value) ATSDR regulation.

Table S3. Frequency of MN, BN, and KL in control and occupationally exposed groups (Mean ± SD, Range.)

Group	Number of subjects	Micronucleus (MN)	Binucleated (BN)	Karyolysis (KL)
**Control**	17	0.35 ± 0.61<br>(0 – 2)	4.18 ± 1.70<br>(2 – 8)	3.47 ± 1.91<br>(1 – 9)
**Miners**	22	2.50 ± 2.65<br>(0 – 9)	12.86 ± 7.27<br>(0 – 26)	17.91 ± 12.86<br>(0 – 59)

Table S4. Concentration (µg L^{-1}) of chemical elements in urine of exposed group (minimum, maximum, mean, median and percentiles 10%, 25%, 75%, and 90%).

Element	Mínimum	Maximum	Mean	Median	Percentile			
					10%	25%	75%	90%
Li	3.88	165.26	30.92	20.70	7.70	14.87	32.75	53.83
Be	0.09	0.61	0.25	0.25	0.15	0.18	0.29	0.34
Al	<LOD*	115.77	33.44	29.42	<LOD*	14.45	39.00	81.76
Ca	11.323.17	26.6427.46	91879.27	92284.76	34688.51	49567.68	127851.51	143940.68
Cr	0.15	0.86	0.38	0.38	0.20	0.24	0.46	0.51
Mn	0.19	4.00	0.72	0.49	0.22	0.33	0.67	1.41
Fe	7.87	58.46	19.65	15.25	8.91	11.86	22.42	29.13
Co	0.23	1.03	0.64	0.65	0.37	0.45	0.86	0.99
Ni	0.66	8.55	3.28	2.91	1.00	2.27	4.36	5.07
Cu	32.69	88.65	59.80	58.64	43.69	48.01	67.02	80.22
Zn	75.06	1357.90	523.60	443.90	124.33	217.20	744.72	940.57
As	5.14	39.02	20.44	20.67	7.37	15.84	22.79	32.32
Se	5.98	37.52	19.72	19.45	8.26	11.21	26.28	30.94
Sr	35.63	563.73	200.04	197.39	74.66	120.32	235.82	304.14
Cd	<LOD*	0.43	0.18	0.14	0.02	0.09	0.29	0.37
Cs	3.83	39.55	22.27	20.56	10.98	15.56	29.12	35.23
Ba	0.39	30.37	6.49	4.28	0.80	1.59	9.87	12.22
Hg	0.22	11.50	3.74	2.47	1.11	1.48	6.33	7.33
Pb	<LOD*	13.88	2.79	1.86	<LOD*	0.19	4.57	5.97
U	<LOD*	<LOD*	<LOD*	<LOD*	<LOD*	<LOD*	<LOD*	<LOD*

* LOD indicates values below the detection limit.

Table S5. Concentration (μg L^{-1}) of chemical elements in urine of control group (minimum, maximum, mean, median and percentiles 10%, 25%, 75%, and 90%).

Element	Mínimum	Maximum	Mean	Median	Percentile			
					10%	25%	75%	90%
Li	2.37	12.33	7.46	7.82	3.82	5.19	9.70	10.97
Be	0.04	0.30	0.13	0.12	0.06	0.09	0.16	0.23
Al	<LOD*	28.50	6.68	3.92	<LOD*	<LOD*	10.99	14.20
Ca	31.406,32	303.139,62	130.072,70	120.290,45	35.240,12	72.249,62	195.966,41	231.884,04
Cr	<LOD*	0.62	0.14	0.12	<LOD*	0.05	0.14	0.27
Mn	<LOD*	0.24	0.03	<LOD*	<LOD*	<LOD*	0.03	0.10
Fe	<LOD*	33.63	13.04	11.27	5.73	8.82	14.04	24.12
Co	0.19	1.61	0.47	0.41	0.20	0.27	0.48	0.74
Ni	0.27	4.90	2.06	1.84	0.49	1.26	3.10	3.67
Cu	15.51	46.44	31.14	33.05	20.14	25.62	36.14	41.54
Zn	148.39	926.08	499.20	443.87	230.10	335.97	647.77	822.18
As	2.11	49.50	13.31	11.13	5.83	6.17	17.22	20.68
Se	10.99	94.18	31.31	26.49	15.24	17.30	33.01	54.36
Sr	64.22	395.94	192.62	161.46	70.74	132.37	220.04	366.96
Cd	<LOD*	0.19	0.06	0.05	0.01	0.04	0.08	0.12
Cs	6.58	38.20	22.73	23.71	14.80	16.84	27.39	33.44
Ba	0.21	18.81	4.73	3.31	1.84	2.21	5.77	9.15
Hg	0.45	2.83	1.33	1.12	0.56	0.91	1.70	2.39
Pb	<LOD*	1.98	0.40	0.18	<LOD*	<LOD*	0.65	1.09
U	<LOD*	<LOD*	<LOD*	<LOD*	<LOD*	<LOD*	<LOD*	<LOD*

* LOD indicates values below the detection limit.

Table S6. Blood concentration (μg L^{-1}) of chemical elements in exposed group (minimum, maximum, mean, median and percentiles 10%, 25%, 75%, and 90%).

Element	Minimum	Maximum	Mean	Median	Percentile			
					10%	25%	75%	90%
Li	0.28	3.67	0.77	0.58	0.33	0.44	0.72	0.89
Be	<LOD*	0.45	0.17	0.11	<LOD*	0.01	0.30	0.37
Al	<LOD*	23.34	3.66	<LOD*	<LOD*	<LOD*	3.76	14.44
Ca	45360.98	62664.78	54522.24	55280.59	48129.52	52137.47	56925.44	58538.90
Cr	<LOD*	3.58	0.28	<LOD*	<LOD*	<LOD*	0.03	0.59
Mn	8.73	17.78	12.85	13.16	8.90	10.08	14.60	16.90
Fe	461817.73	721983.16	587174.01	578055.21	546741.57	556251.12	613535.91	665096.52
Co	0.30	0.68	0.44	0.39	0.33	0.35	0.49	0.64
Ni	<LOD*	281.94	15.27	0.04	<LOD*	<LOD*	5.49	10.25
Cu	833.98	1403.48	1062.64	1046.61	894.72	973.90	1136.98	1215.32
Zn	5377.24	8640.76	7128.18	6973.87	6105.91	6478.32	7942.23	8395.99
As	0.68	2.83	1.45	1.39	0.98	1.18	1.71	1.87
Se	37.98	65.80	53.79	54.44	44.87	47.93	58.17	63.23
Sr	6.35	19.93	11.18	10.69	8.45	9.10	12.63	14.61
Cd	<LOD*	0.38	0.14	0.13	0.05	0.08	0.20	0.23
Cs	4.00	11.64	7.46	7.22	4.88	6.05	8.84	10.38
Ba	0.27	6.50	1.46	0.91	0.49	0.60	1.78	2.78
Hg	0.22	4.09	0.87	0.64	0.35	0.40	1.01	1.11
Pb	18.60	204.26	74.26	60.11	29.74	37.78	99.27	126.80
U	<LOD*	<LOD*	<LOD*	<LOD*	<LOD*	<LOD*	<LOD*	<LOD*

* LOD indicates values below the detection limit.

Table S7. Blood concentration (μg L^{-1}) of chemical elements in control group (minimum, maximum, mean, median and percentiles 10%, 25%, 75%, and 90%).

Element	Minimum	Maximum	Mean	Median	Percentile			
					10%	25%	75%	90%
Li	0.02	0.61	0.34	0.36	0.17	0.21	0.47	0.56
Be	<LOD*	0.62	0.18	0.13	0.05	0.09	0.18	0.41
Al	<LOD*	123.43	7.42	<LOD*	<LOD*	<LOD*	<LOD*	1.07
Ca	47,545.59	81,846.64	55,508.42	53,028.06	50,464.84	51,197.95	55,703.05	63,491.59
Cr	<LOD*	10.73	0.77	<LOD*	<LOD*	<LOD*	0.25	0.90
Mn	6.34	18.30	10.59	10.56	6.75	9.12	11.50	13.33
Fe	507058.49	859,988.76	580,573.83	559,489.16	533,547.60	544,316.16	585,012.80	607,851.48
Co	0.25	0.59	0.36	0.34	0.30	0.32	0.43	0.46
Ni	<LOD*	32.16	3.34	<LOD*	<LOD*	<LOD*	1.17	9.06
Cu	639.90	2,512.27	1,109.77	983.98	841.32	919.46	1,029.12	1,634.38
Zn	5,935.96	10,897.07	7,213.15	7,072.17	6,095.39	6,175.54	7,704.23	8,285.53
As	1.26	9.86	4.18	2.75	1.56	1.96	4.81	8.76
Se	53.30	93.70	72.82	70.86	58.71	66.53	79.10	89.32
Sr	4.80	13.84	7.83	7.33	5.95	6.34	9.48	9.89
Cd	<LOD*	0.52	0.12	0.08	0.03	0.04	0.14	0.29
Cs	7.63	18.12	13.62	14.36	8.77	11.21	16.42	17.43
Ba	0.13	2.73	1.06	0.79	0.41	0.61	1.18	2.67
Hg	0.29	3.32	0.96	0.64	0.32	0.39	1.39	1.73
Pb	7.04	41.76	17.02	14.59	8.03	10.15	21.66	26.08
U	<LOD*	<LOD*	<LOD*	<LOD*	<LOD*	<LOD*	<LOD*	<LOD*

* LOD indicates values below the detection limit.

Table S8. Concentrations (μg g^{-1} creatinine) of chemical elements in urinary.

Element	Minimum	Maximum	Mean	Median	Percentile			
					10%	25%	75%	90%
Li	0.66	23.85	4.84	3.43	2.03	2.52	4.78	7.92
Be	0.01	0.14	0.05	0.04	0.02	0.03	0.06	0.07
Al	0.00	82.87	8.13	4.71	0.00	1.98	7.21	10.50
Ca	1391.83	53115.92	16706.70	11561.65	5021.65	8455.30	22896.53	32798.04
Cr	0.01	0.28	0.07	0.06	0.03	0.04	0.08	0.13
Mn	0.02	2.86	0.23	0.07	0.02	0.05	0.15	0.24
Fe	1.00	20.68	4.14	2.49	1.04	1.75	4.66	9.05
Co	0.03	0.23	0.11	0.11	0.05	0.08	0.15	0.17
Ni	0.07	1.81	0.56	0.51	0.27	0.45	0.64	0.87
Cu	3.49	31.27	11.17	9.66	5.95	6.74	13.80	17.55
Zn	11.75	440.28	91.40	67.76	31.11	48.22	110.71	144.63
As	1.62	11.58	3.43	2.94	1.86	2.39	3.92	4.82
Se	1.52	4.61	3.05	3.19	2.07	2.41	3.73	3.86
Sr	4.38	80.37	35.56	28.79	16.10	19.98	54.90	67.08
Cd	0.00	0.10	0.03	0.02	0.00	0.01	0.04	0.07
Cs	1.36	6.89	3.59	3.44	2.19	2.68	4.26	4.94
Ba	0.05	5.57	1.21	0.61	0.12	0.30	1.68	2.83
Hg	0.03	2.43	0.74	0.35	0.10	0.28	0.96	1.92
Pb	0.00	2.00	0.48	0.24	0.00	0.03	0.75	1.26
U	0.00	0.00	0.00	0.00	0.00	0.00	0.00	0.00

Table S9. Relation (µg L^{-1}) between elements in miners' urine and age groups.

Age group (years)	n	Li	Be	Al	Ca	Cr	Mn	Fe	Co	Ni	Cu	Zn	As	Se	Sr	Cd	Cs	Ba	Hg	Pb	U
20-30	1	7.21	0.292	12.68	43736.14	**0.52**	0.22	11.36	0.318	0.73	54.27	172.84	17.73	**31.02**	73.64	0.10	**39.53**	1.076	1.11	0.00	0.00
30-40	1	36.52	0.089	0.00	**144433.24**	0.15	0.23	15.64	0.699	3.82	50.66	546.09	**32.16**	24.44	**237.87**	0.07	27.28	5.726	**11.50**	2.38	0.00
40-50	3	12.34	0.259	21.06	68027.37	0.29	0.50	17.09	0.643	3.35	58.29	372.67	25.31	25.06	141.67	0.17	25.49	2.065	4.10	1.47	0.00
50-60	12	27.41	**0.294**	**44.97**	96837.02	0.42	0.**88**	18.31	0.639	3.48	**64.49**	**630.30**	20.14	18.30	222.96	0.18	19.75	7.545	3.46	2.37	0.00
60 -70	3	**78.36**	0.144	26.64	100571.70	0.42	0.75	**34.16**	**0.737**	**3.88**	50.26	626.55	17.53	21.77	235.45	**0.27**	27.33	**9.171**	3.27	**6.99**	0.00
70-80	2	17.74	0.204	20.11	82666.62	0.27	0.49	15.85	0.668	2.03	55.58	119.50	14.77	9.09	141.17	0.14	13.84	5.820	3.03	2.62	0.00

*Bold indicates the maximum value in each age group

Table S10. Relation (µg L^{-1}) between elements in miners' blood and age groups.

Age group (years)	n	Li	Be	Al	Ca	Cr	Mn	Fe	Co	Ni	Cu	Zn	As	Se	Sr	Cd	Cs	Ba	Hg	Pb	U
20-30	1	0.28	0.00	0.00	52895.05	0.00	**13.16**	569634.89	0.473	0.00	980.42	6798.32	**1.89**	**62.98**	9.04	0.00	6.19	0.707	**4.09**	**127.83**	0.00
30-40	1	0.53	0.07	0.00	56935.92	0.31	10.06	583184.53	0.427	0.00	972.83	6610.94	1.30	49.63	8.43	0.08	**11.34**	1.769	2.57	41.93	0.00
40-50	3	0.46	0.27	0.00	54593.15	0.00	11.72	579976.52	0.365	1.95	1066.04	6934.40	1.38	55.51	9.19	0.13	6.17	0.725	1.06	68.13	0.00
50-60	12	0.72	**0.19**	5.03	53823.18	0.09	13.57	**609528.76**	0.430	**26.08**	1024.05	**7476.80**	1.44	53.54	11.87	0.15	7.11	1.634	0.58	67.85	0.00
60 -70	3	**1.67**	0.18	**6.75**	55196.04	0.41	11.81	582534.39	**0.489**	2.24	**1231.44**	6488.31	1.32	58.72	**12.21**	**0.20**	10.25	**1.784**	0.58	112.07	0.00
70-80	2	0.56	0.00	0.00	**57206.33**	**1.79**	13.09	481565.41	0.484	5.23	1121.91	6710.40	1.62	42.73	10.94	0.13	6.03	1.240	0.31	54.60	0.00

*Bold indicates the maximum value in each age group

Table S11. Relation (μg L^{-1}) between elements in miners' urine and working time.

**Working time (years)**	**n**	**Li**	**Be**	**Al**	**Ca**	**Cr**	**Mn**	**Fe**	**Co**	**Ni**	**Cu**	**Zn**	**As**	**Se**	**Sr**	**Cd**	**Cs**	**Ba**	**Hg**	**Pb**	**U**
0 - 5	1	7.21	0.29	12.68	43736.14	**0.52**	0.22	11.36	0.32	0.73	54.27	172.84	17.73	**31.02**	73.64	0.10	**39.53**	1.08	1.11	0.00	0.00
5 – 10	3	29.25	0.19	25.54	123470.33	0.31	0.52	12.26	0.62	**4.34**	54.46	646.69	21.41	19.08	255.02	0.07	21.89	6.25	**8.01**	0.95	0.00
10 – 15	3	28.85	0.23	15.96	**140695.56**	0.49	0.43	21.01	0.72	3.46	54.61	626.34	20.89	23.95	**294.30**	0.22	24.14	7.08	4.04	3.53	0.00
15 – 20	3	13.62	0.23	31.74	92345.21	0.39	0.75	14.25	0.64	2.97	67.36	**771.14**	14.09	15.98	185.53	**0.25**	19.21	4.89	4.54	2.79	0.00
20 – 30	4	**54.34**	**0.30**	38.11	87252.03	0.33	0.79	**34.17**	0.58	3.45	55.99	429.82	23.02	18.17	220.44	0.20	23.51	**12.56**	4.42	**6.15**	0.00
30 – 40	6	36.85	0.29	39.19	68573.45	0.37	0.48	15.03	**0.83**	3.76	**70.11**	549.92	**26.35**	22.93	166.90	0.19	23.39	4.27	1.25	1.70	0.00
40 -50	2	9.72	0.17	**57.89**	73812.89	0.36	**2.20**	25.71	0.31	1.36	43.72	97.50	6.26	7.75	119.70	0.14	10.16	5.55	3.13	2.42	0.00

*Bold indicates the maximum value in each age group

Table S12. Relation (μg L^{-1}) between elements in miners' blood and working time.

**Working time (years)**	**N**	Li	Be	Al	Ca	Cr	Mn	Fe	Co	Ni	Cu	Zn	As	Se	Sr	Cd	Cs	Ba	Hg	Pb	U
0 - 5	1	0.28	0.000	0.00	52895.05	0.00	13.16	569634.89	0.473	0.00	980.42	6798.32	**1.89**	**62.98**	9.04	0.000	6.19	0.71	**4.09**	**127.83**	0.00
5 - 10	3	0.55	0.150	0.00	51708.96	0.10	11.27	604320.51	0.407	0.00	999.95	7394.31	1.41	54.84	9.81	0.108	**9.26**	1.07	1.06	35.88	0.00
10 - 15	3	0.60	**0.231**	1.57	55434.99	0.02	11.22	588018.45	0.494	0.00	1112.06	**7661.38**	1.11	60.70	11.53	0.163	7.93	0.84	0.52	71.98	0.00
15 - 20	3	0.52	0.110	0.00	**58011.23**	0.18	12.00	598673.46	0.353	5.81	1054.13	7202.76	1.29	52.24	11.14	0.064	6.51	0.77	0.67	63.24	0.00
20 - 30	4	**1.30**	0.227	1.74	53973.27	0.15	12.33	**605198.10**	0.412	2.09	984.13	7323.04	1.44	53.11	12.03	0.107	7.38	1.76	0.64	109.56	0.00
30 - 40	6	0.92	0.175	**11.49**	53807.86	**0.79**	**15.78**	570567.91	**0.505**	**51.63**	1102.18	6572.97	1.59	49.81	**12.23**	**0.207**	7.04	**2.45**	0.85	71.85	0.00
40 -50	2	0.50	0.160	0.00	56194.24	0.00	11.10	565478.05	0.343	0.20	**1174.87**	7258.12	1.64	52.85	9.01	0.205	7.56	0.81	0.29	61.61	0.00

*Bold indicates the maximum value in each age group
